# Comparing Methodology and Recruitment Strategies in Two Studies on Chinese and Korean American Dementia Caregivers

**DOI:** 10.1093/geroni/igaf122.1735

**Published:** 2025-12-31

**Authors:** Eunjung Ko, Karen Rose, Mary Mittelman, Bei Wu

**Affiliations:** New York University, New York, New York, United States; The Ohio State University, The Ohio State University, Ohio, United States; New York University Grossman School of Medicine, New York, New York, United States; NYU Shanghai, Shanghai, China (People’s Republic)

## Abstract

Recruiting individuals from East Asian cultures for research presents unique challenges, which vary based on study methodologies, recruitment strategies, and cultural sensitivities. We compared methodologies and recruitment strategies in two studies targeting Chinese and Korean American dementia caregivers—a completed qualitative study and an ongoing psychosocial randomized controlled trial (RCT). The qualitative study explored participants’ help-seeking behaviors for their mental health, while the ongoing year-long RCT is implementing a culturally adapted, validated intervention. Both used purposive sampling and offered participant compensation. However, the former study included first or second-generation foreign-born individuals fluent in English, regardless of age or health risks, whereas the latter is targeting individuals aged 50 or older with one or more cardiometabolic disease risk factors, regardless of language proficiency. In the qualitative study, social media, a research registry, and word-of-mouth were used to recruit 20 participants across the US over eight months. In contrast, community engagement, social service agencies, social media, and word-of-mouth are being used in the RCT; over 170 people living within the New York metropolitan area have been recruited since 2023. These different recruitment strategies appeared to influence participants’ characteristics. All participants in the qualitative study had tertiary degrees, with 40% earning over $100,000 annually. However, over a third of RCT participants had a high school education or lower, most reported low levels of English proficiency, and fewer than 8% earned $100,000 or more. This comparison indicates that recruitment strategies and study methodology can influence participant demographics, suggesting the need for tailored recruitment approaches.

